# Aquaporin 1 affects pregnancy outcome and regulates aquaporin 8 and 9 expressions in the placenta

**DOI:** 10.1007/s00441-020-03221-w

**Published:** 2020-06-15

**Authors:** Hui Luo, Yi Liu, Yizuo Song, Ying Hua, Xueqiong Zhu

**Affiliations:** Department of Obstetrics and Gynecology, The Second Affiliated Hospital of Wenzhou Medical University, No. 109 Xueyuan Xi Road Wenzhou, Zhejiang, 325027 China

**Keywords:** Aquaporin, Pregnancy outcome, Amniotic fluid, Placenta, Foetal membrane

## Abstract

To explore the effects of aquaporin (AQP) 1 on pregnancy outcome and the association between expression of AQP1 and other AQPs in the placenta and foetal membranes, the rate of copulatory plugs and pregnancy, amniotic fluid (AF) volume, osmolality and composition were determined in *AQP1*-knockout (*AQP1*^−/−^) mice at different gestational days (GD). The expression and location of AQP1 and other AQPs in the placenta and foetal membranes of *AQP1*^−/−^ mice, AQP1-siRNA transfected WISH cells and oligohydramnios patients were also detected. Compared to control mice, *AQP1*^−/−^ mice exhibited reduced copulation plug and successful pregnancy rates, but these effects were accompanied by a larger AF volume and lower AF osmolality at late gestation. AQP9 expression was significantly decreased in the placenta and foetal membranes of *AQP1*^−/−^ mice, while AQP8 level was elevated in the foetal membranes of *AQP1*^−/−^ mice. Moreover, AQP9 expression was suppressed in WISH cells after AQP1 downregulation. Furthermore, AQP9 expression was associated with AQP1 level in the placenta and foetal membranes in oligohydramnios. AQP1 may play a critical role in regulating pregnancy outcome and maternal-foetal fluid homeostasis. Changes in AQP1 expression may lead to compensatory alterations in AQP8 and AQP9 expression in the placenta.

## Introduction

The homeostasis of amniotic fluid (AF) exchange between matrix and foetus plays a vital role in a successful pregnancy, as either polyhydramnios (excess AF) or oligohydramnios (insufficient AF) may increase foetal morbidity and mortality during the perinatal stage (Ducza et al. 2017). However, the underlying cellular and molecular mechanisms of solute and water transport between the foetal and maternal compartments remain elusive and require investigation.

During the latter period of gestation, AF is mainly derived from foetal urine and a portion of the lung fluid (Brace and Cheung 2014). The major mechanisms by which fluid is removed from the amniotic cavity are foetal swallowing and intramembranous absorption (Harman 2008), which involves in the transfer of amniotic water and solutes between the amnion and chorion and the entrance of amniotic water and solutes into underlying foetal blood vessels (Brace and Cheung 2014). This process primarily regulates AF volume (Anderson et al. 2013). Aquaporins (AQPs), small integral membrane proteins with a monomeric size ranging from 26 to 34 kDa (Gonen and Walz 2006, Carbrey and Agre 2009), act as water channels and were reported to be specifically crucial in governing the AF volume (Hua et al. 2013). To date, a total of 13 AQP proteins (AQP0 to AQP12) have been identified in mammals (Verkman 2011). Each monomeric AQP contains a pore that osmotically facilitates the movement of water across the cell membrane.

AQP1, expressed mainly in the amnion epithelium, chorion cytotrophoblasts and placental vessels (Zhu et al. 2010), is important for the osmotic movement of water across the barrier between the endothelium and epithelium (Mobasheri and Marples 2004). Mann et al. showed that transgenic *AQP1*-knockout (AQP1^−/−^) mice developed an increased AF volume and reduced AF osmolality (Mann et al. 2005), suggesting that the loss of AQP1 causes idiopathic polyhydramnios. However, the authors did not observe the changes in AF volume and osmolality in mice with *AQP1* depletion during different gestational days. Intriguingly, this group subsequently discovered that in pregnancies with polyhydramnios, AQP1 expression was enhanced in all regions of the foetal membrane, demonstrating that alterations in AQP1 expression might be a compensatory response but not the primary aetiolgy of idiopathic polyhydramnios (Mann et al. 2006). Additionally, AQP3, AQP8 and AQP9 expressions have been detected in both the placenta and foetal membranes, indicating that these molecules could play a vital role in maternal-foetal fluid exchange (Prat et al. 2012; Zhang et al. 2012b). Our previous studies indicated that the expression of AQP1, AQP3, AQP8 and AQP9 in foetal membranes was dramatically reduced in pregnant women diagnosed with isolated oligohydramnios (Zhu et al. 2009, Jiang et al. 2012). However, an association between expression of AQP1 and other AQPs in patients with a normal AF volume and patients with isolated oligohydramnios has not been established.

Recently, the *AQP*^*−/−*^ mouse model was characterized as a powerful approach for investigating the physiological role and function of AQPs. Using *AQP8*^−/−^ mice, Su et al. showed that the mRNA expression of AQP1, AQP11 and AQP12 but not AQP5, was significantly decreased in neonatal mouse ovaries (Su et al. 2013). Similarly, another study dissected starved *AQP7*^*−/−*^ mice and found that AQP1 expression was upregulated in the capillaries of white adipose tissue in response to prolonged starvation (Skowronski et al. 2016). However, the effects of *AQP1* gene knockout on pregnancy rate and outcome in female mice as well as the expression of other AQP proteins (AQP3, AQP8 and AQP9) in both the placenta and foetal membranes of pregnant *AQP1*^−/−^ mice have not been investigated until now.

Therefore, a transgenic *AQP1*^−/−^ mouse model was generated to observe the effects of AQP1 depletion on the following gestation-related parameters on different gestational days: maternal pregnancy rate, foetal development and AF volume and osmolality. In addition, both the placenta and foetal membranes were collected to measure the mRNA and protein expression profiles of AQP3, AQP8 and AQP9 in mice after *AQP1* depletion. Moreover, the correlation among the protein expression of AQP1 and other AQPs in the placenta and foetal membranes of patients with isolated oligohydramnios were also explored using an immunohistochemical method. Finally, after using small interfering RNA (siRNA) to interfere with AQP1 expression in human amnion epithelial WISH cells, the mRNA and protein expression levels of AQP3, AQP8 and AQP9 were examined.

## Materials and methods

### Transgenic *AQP1*^−/−^ mice

*AQP1*^−/−^ mice were established through intercrossing *AQP1* heterozygous mice overnight (Ma et al. 1998). *AQP1* heterozygous (*AQP1*^+/−^) mice were donated by Prof. Yuanlin Song from Zhongshan Affiliated Hospital of Fudan University. *AQP1*^−/−^ and wild-type *AQP1* (*AQP1*^+/+^) mice were mated separately. Gestational day 0.5 (0.5 GD) was defined as the day when a copulation plug was observed, with normal pregnant mice delivering foetuses at term on 19 GD to 20 GD. Meanwhile, the copulation plug and pregnancy rates were defined as the number of female mice with a copulation plug/number of total mice and the number of pregnancy/number of total mice, respectively. These two rates were recorded. Concept uses were collected from pregnant *AQP1*^−/−^ and *AQP1*^+/+^ mice at 9.5 GD (early pregnancy), 13.5 GD (middle pregnancy) and 16.5 GD (late pregnancy).

All mice, which were 6–8 weeks old, were kept under a12-h light/dark cycle at a temperature of 23 ± 1 °C. A standard diet and deionized water were freely accessible to the mice. All animal experiments were carried out according to the Guide for the Care and Use of Laboratory Animals published by the United States National Institutes of Health. Protocols were approved by the Animal Care and Use Committee of Wenzhou Medical University.

### Detection of AF volume; foetus, amnion and placenta weights; and placental area

Five pregnant *AQP1*^−/−^ pregnant mice and five pregnant *AQP1*^+/+^ mice at each gestational day (9.5GD, 13.5GD and 16.5GD) were used for this study. At the appropriate gestational age, caesarean section was performed on the two groups of mice, and each gestational sac was carefully separated. The number of embryos per pregnant mouse and macroscopic atrophy were recorded at 9.5 GD, 13.5 GD and 16.5 GD. Each gestational sac was weighed and then ruptured. After the AF had been collected into an Eppendorf tube, the foetus, foetal membrane (including both the amnion and chorion) and placenta were weighed. These tissues were collected from foetuses on each gestational day (GD) and stored at – 80 °C for further study. The placental area was calculated after measuring the diameter by using the formula: *S* = π × (1/2 × diameter)^2^. The AF mass was estimated by determining the difference in weight before and after-rupture.

### Determination of AF osmolality and composition

AF collected from each sac was centrifuged at 3000 rpm for 10 min to remove cellular debris. Subsequently, the AF from each litter of the same genotype was mixed and used to determine the AF osmolality and composition. The cryoscopic method was used to measure AF osmolality with automatic freezing point osmometer (FM-8P, China). Glucose and total protein in the AF were detected by the biuret method, and urea and creatinine levels were examined by enzymic methods. The electrode method was conducted with o-cresolphthalein complexone on an automatic biochemical analyser (ADVIA2400, USA) to determine the concentrations of electrolytes (Na^+^, K^+^, Cl^−^) and another ion (Ca^2+^).

### Cell culture

Human amniotic epithelial cells (WISH cells) were purchased from the Type Culture Collection of the Chinese Academy of Medical Sciences (Shanghai, China). The cells were cultured in Dulbecco’s modified eagle’s medium (DMEM) (Gibco, USA) supplemented with 10% foetal bovine serum (Gibco, USA) and a100 × Pen-Strep solution (Invitrogen, USA). The cells were incubated at 37 °C in a humidified atmosphere containing 5% CO_2_. The culture medium was replaced once every 2 days.

### AQP1 siRNA transfection

Both AQP1 siRNA and control siRNA were purchased from GenePharma (Shanghai, China). The sequence of AQP1 siRNA was 5′-GCU GUA CUC AUC UAC GAC UTT, and the antisense sequence was 5′-AGU CGU AGA UGA GUA CAG CTT. The control siRNA sequence was 5′-UGA CC UCA ACU ACA UGG UGT T, and the antisense sequence was 5′-AAC CAU GUA GUU GAG GUC ATT. WISH cells were cultured to a confluence of 50% and subsequently transfected with either AQP1 siRNA or control siRNA at 40 nM using Lipofectamine 2000 (Invitrogen, USA) in DMEM without foetal bovine serum following the manufacturer’s protocol. The media were changed after 6 h of incubation and transfected cells were cultured for another 48 h for subsequent studies.

### Quantitative reverse transcription polymerase chain reaction analyses

Total RNA was extracted from the foetal membrane, placenta and WISH cells using TRIzol in accordance with the manufacturer’s protocol. The absorbance of the RNA at 260 nm and 280 nm was quantified by spectrometry. From 1 μg of total RNA, cDNA was synthesized using a reverse transcription kit following the manufacturer’s instructions (Thermo Scientific, Wilmington, USA). Each quantitative RT-PCR sample contained 5 μL of SYBRGreen Master Mix (TransStart TipTop), 1 μL of cDNA, 0.5 μL of primer pairs complementary to AQPs or GAPDH, and diethyl pyrocarbonate (DEPC) H_2_O at a volume of 10 μL, and reactions were carried out on a Light Cycler480 (Roche, France). The cycling parameters were as follows: 95 °C for 5 min, followed by 40 cycles of denaturation at 95 °C for 20 s, annealing at 58 °C for 1 min and extension at 72 °C for 20 s. Table 1 provides sequences of the primer sets for AQP1, AQP3, AQP8, AQP9 and GAPDH in detail. All transcripts were independently quantified three times. Relative gene expression levels were determined by using the comparative cycle threshold (2^△△^Ct) method, and GAPDH was used as a normalization reference.

**Table 1:** Primers of AQPs and GAPDH for murine placentas and foetal membranes

Gene	Forward primer (5′-3′)	Reverse primer (3′-5′)
AQP1	AGCAGCGACTTCACAGAC	CTATTTGGGCTTCATCTCC
AQP3	ATTGTCTCCCCACTCCTG	TCACATTCTCTTCCTCGG
AQP8	TAAGCCCCATTCTCCATT	AGTAGCCAGCCATCACAG
AQP9	CTTCCACCATCCTTCCAC	TGAGCAATAGAGCCACATC
GAPDH	AAGAAGGTGGTGAAGCAGG	GAAGGTGGAAGAGTGGGAGT

*AQP*, aquaporin; *GAPDH*, glyceraldehyde-3-phosphate dehydrogenase

### Western blotting

Placental and foetal membrane tissues and WISH cells were separately homogenized and lysed in radio immunoprecipitation assay (RIPA) buffer (Beyotime, China) supplemented with protease inhibitor cocktail (Thermo, USA). The lysate concentrations were determined by bicinchoninic acid (BCA) protein assay. Then, samples containing 40 μg of protein were separated by 12% sodium dodecyl sulphate-polyacrylamide gel electrophoresis (SDS-PAGE), and the proteins were then transferred onto a polyvinylidene fluoride (PVDF) membrane (Millipore, USA). The membranes were blocked for 2 h with 5% skimmed milk in Tris-buffered saline with 0.1% Tween-20 (TBS-T, pH 7.4) at room temperature and then incubated at 4 °C overnight with a primary antibody. Membranes were incubated with the following primary antibodies at 4  C overnight: monoclonal mouse anti-AQP1 antibody (1:1000, sc-25287, Santa Cruz, USA), polyclonal rabbit anti-AQP3 antibody (1:500, ab125219, Abcam, USA), polyclonal rabbit anti-AQP8antibody (1:500, ab203682, Abcam), monoclonal mouse anti-AQP9 antibody (1:1000, sc-74409, Santa Cruz, USA), antibody against endogenous α-Tubulin was used as an internal control (1:5000, AF0001, Beyotime, China). The membranes were rinsed and probed with horseradish-peroxidase-conjugated secondary antibody (Biosharp, China) diluted 1:5000 for 2 h at room temperature. Finally, immunoreactive bands containing target proteins were visualized with enhanced chemiluminescence (ECL) substrate (Thermo, USA), and images were captured with an Amersham Imager 600 system (General Electric Company, USA).

### Case selection and diagnostic criteria for oligohydramnios

To detect correlations between the expression of AQP1 and other AQPs when AQP1 and other AQPs were decreased, a total of 30 patients with singleton pregnancies who received elective caesarean delivery were enrolled in this study; among these patients, 15 patients were identified as having oligohydramnios, while the other 15 had a normal AF volume. The gestational age of the pregnant women ranged from 37 and 40 weeks. Women were excluded when at least one of the following conditions was met: foetal abnormalities; a foetus small for the gestational age; premature rupture of the membranes; restricted foetal growth; treatment with several medications (including those used for cervical ripening, augmentation or labour induction); and diagnosis with complications that might influence the AF, such as hypertension, diabetes, cardiovascular disorders and autoimmune diseases. Ethical approval was received from the Ethical Committee of the Second Affiliated Hospital of Wenzhou Medical University. All subjects agreed to the study and provided written informed consent before starting the study.

Before delivery, oligohydramnios was defined as an AF index (AFI) < 5 cm using ultrasound measurement, as a normal AFI ranges from to 8 to 18 cm (Phelan et al. 1987; Zhang et al. 2004). During delivery, the AF volume (AFV) was measured. An AFV less than 300 mL indicated oligohydramnios, while an AFV from 300 to 2000 mL indicated a normal AFV (Zhu et al. 2009).

### Immunohistochemical staining

The placenta and foetal membranes were isolated from patients in the normal group and patients with oligohydramnios and fixed in a 4% paraformaldehyde solution. These tissues were embedded in paraffin and sliced into sections at a thickness of 5 μm. Subsequently, the sections were dried, deparaffined and rehydrated in an ethanol gradient. Then, tissue slides were washed with phosphate-buffered saline (PBS), placed in a 10 mM citrate solution (pH 6.0) and heated at 100 °C in a microwave oven for antigen retrieval. Endogenous peroxidase activity was blocked with 3% H_2_O_2_ at room temperature, and nonspecific binding was prevented via incubation with a 5% blocking bovine serum albumin solution. Tissue sections were then incubated with primary antibodies overnight at 4 °C. The following antibodies were used for immunohistochemical (IHC) staining: monoclonal anti-AQP1 mouse antibody (1:100, sc-25287, Santa Cruz, USA), polyclonal rabbit anti-AQP3 antibody (1:100, ab125219, Abcam, USA), polyclonal rabbit anti-AQP8antibody (1:300, ab203682, Abcam, USA) and monoclonal mouse anti-AQP9 antibody (1:200, GTX47915, GeneTex, USA). The tissues were then incubated with secondary antibody for 20 min at room temperature, followed by incubation with diaminobenzidine as a chromogen for the appropriate duration. After washing with PBS for three times, the tissue sections were counterstained in haematoxylin, dehydrated, cleared and mounted in dibutyl phthalate polystyrene xylene. At least 10 representative staining fields were chosen under a microscope (Olympus-FM10, Japan), and all immunopositive cells in these fields were analysed at random; the proportion of AQP-positive cells was determined for each case (Craciun and Domsa 2017).

### Statistical analyses

SPSS 19.0 (Chicago, IL) was used for statistical analysis. Differences in the copulation plug and pregnancy rates were analysed by chi-square test. Quantitative data are expressed as the mean ± standard deviation (SD). The statistical significance of differences in AF volume, osmolality and composition; feotal weight; amnion weight; placental area; and weight between the *AQP1*^−/−^ and *AQP1*^+/+^ mice were assessed by analysis of variance (ANOVA). Multiple comparisons were carried out by using a post hoc least significant difference (LSD) test. Correlation analysis of the expression of AQP1 and other AQPs was conducted using Pearson correlation analysis for normally distributed data; otherwise, the Spearman correlation test was used. A two-sided *P* < 0.05 indicated a statistically significant difference.

## Results

### AQP1 deficiency reduces fertility in mice

First, we explored fertility in mice after *AQP1* depletion. We found that pregnant *AQP1*^−/−^ mice exhibited a reduced copulation plug rate (50%, 30/60) compared with that in pregnant *AQP1*^+/+^ mice (90%, 54/60), suggesting that AQP1 depletion led to a decrease in the copulation plug rate (χ^2^ = 4.09, *P* = 0.043). The pregnancy success rate was higher in *AQP1*^+/+^ mice (94.4%, 51/54) than in wild-type controls (33.3%, 10/30, χ^2^ = 6.60, *P* = 0.01). However, the numbers of macroscopic atrophic embryos per pregnancy recorded in *AQP1*^−/−^ mice and *AQP1*^+/+^ mice at each gestational age were no different. These results show that AQP1 deficiency reduces reproductive performance in female mice.

### *AQP1* knockout increases AF weight and decreases AF osmolality in mice

We further determined whether AQP1 depletion affects the volume, osmolality and composition of the AF in mice at different stages of pregnancy. The AF volume was not determined, and the placenta and foetal membrane were not dissected as they had not yet formed in the gestational sac at 9.5 GD. There was no significant difference in the AFV between *AQP1*^−/−^ and *AQP1*^+/+^ mice at 13.5 GD (*P* > 0.05, Table 2). Intriguingly, the AF weight was dramatically higher in *AQP1*^−/−^ mice than in *AQP1*^+/+^mice at 16.5 GD (*P* < 0.05, Table 3).

**Table 2:** Changes in AF, osmolality, composition concentration and foetus, foetal membrane and placenta weights as well as placenta areas among the AQP1 homozygote and wild-type groups at 13.5 GD (mean ± SD)

Parameters	AQP1^+/+^	AQP1^−/−^
AF (g)	135 ± 19.2 (*n* = 5)	130 ± 29.1 (*n* = 5)
Osmolality (mOsm/L)	301 ± 24.7 (*n* = 5)	302 ± 29.0 (*n* = 5)
Total protein (g/L)	3.40 ± 0.990 (*n* = 5)	3.62 ± 0.420 (*n* = 5)
Glucose (g/L)	2.37 ± 0.862 (*n* = 5)	3.35 ± 0.861 (*n* = 5)
Urea (mmol/L)	8.04 ± 1.61 (*n* = 5)	7.31 ± 1.92 (*n* = 5)
Creatinine (mmol/L)	9.83 ± 1.45 (*n* = 5)	10.23 ± 3.26 (*n* = 5)
Na^+^ (mmol/L)	141 ± 8.34 (*n* = 5)	140.5 ± 10.7 (*n* = 5)
K^+^ (mmol/L)	6.61 ± 0.792 (*n* = 5)	7.58 ± 1.27 (*n* = 5)
Cl^−^ (mmol/L)	102 ± 8.25 (*n* = 5)	105 ± 9.51 (*n* = 5)
Ca^2+^ (mmol/L)	1.81 ± 0.641 (*n* = 5)	1.93 ± 0.572 (*n* = 5)
Foetus weight (mg)	140 ± 19.5 (*n* = 41)	132 ± 36.8 (*n* = 37)
Foetal membrane weight (mg)	15.9 ± 3.27 (*n* = 41)	16.1 ± 4.33 (*n* = 37)
Placenta weight (mg)	65.4 ± 10.3 (*n* = 41)	74.3 ± 18.0* (*n* = 37)
Placental area (mm^2^)	0.453 ± 0.0201 (*n* = 41)	0.422 ± 0.0312 (*n* = 37)

Data were analysed by ANOVA. *P < 0.05 versus wild type (*AQP1*^+/+^group)

*GD*, gestational day; *SD*, standard deviation; *ANOVA*, analysis of variance

**Table 3:** Changes in AF, osmolality, composition concentration and foetus, foetal membrane and placenta weights as well as placenta areas among the AQP1 homozygote and wild-type groups at 16.5 GD (mean ± SD)

Parameters	AQP1^+/+^	AQP1^−/−^
AF (g)	183 ± 25.5 (*n* = 5)	196 ± 23.3* (*n* = 5)
Osmolality (mOsm/L)	400 ± 16.5 (*n* = 5)	312 ± 6.01* (*n* = 5)
Total protein (g/L)	2.86 ± 0.412 (*n* = 5)	2.69 ± 0.642 (*n* = 5)
Glucose (g/L)	1.83 ± 0.621 (*n* = 5)	1.86 ± 0.761 (*n* = 5)
Urea (mmol/L)	7.43 ± 1.10 (*n* = 5)	7.07 ± 1.17 (*n* = 5)
Creatinine (mmol/L)	13.0 ± 3.36 (*n* = 5)	12.7 ± 5.57 (*n* = 5)
Na^+^ (mmol/L)	146 ± 4.28 (*n* = 5)	142.4 ± 4.95 (*n* = 5)
K^+^ (mmol/L)	7.82 ± 1.84 (*n* = 5)	8.18 ± 1.81 (*n* = 5)
Cl^−^ (mmol/L)	109 ± 6.03 (*n* = 5)	108.6 ± 4.70 (*n* = 5)
Ca^2+^ (mmol/L)	1.73 ± 0.472 (*n* = 5)	1.40 ± 0.361(*n* = 5)
Foetus weight (mg)	556 ± 121 (*n* = 42)	656 ± 115* (*n* = 40)
Foetal membrane weight (mg)	30.1 ± 7.22 (*n* = 42)	31.2 ± 6.58 (*n* = 40)
Placenta weight (mg)	79.2 ± 11.6 (*n* = 42)	79.9 ± 9.91 (*n* = 40)
Placental area (mm^2^)	0.412 ± 0.0323 (*n* = 42)	0.451 ± 0.0412 (*n* = 40)

Data were analysed by ANOVA. *P < 0.05 versus wild type (*AQP1*^+/+^group)

*GD*, gestational day; *SD*, standard deviation; *ANOVA*, analysis of variance

No significant difference in AF osmolality or total protein, glucose, urea, creatinine or ion (Na^+^, K^+^, Cl^−^ and Ca^2+^) concentrations was observed between the two groups at 13.5 GD (Table 2). Consistently, there was also no significant difference in the AF composition between the two groups at 16.5 GD (Table 3), but the *AQP1*^−/−^ embryos had a lower AF osmolality than their *AQP1*^+/+^counterparts at 16.5 GD (*P* < 0.05, Table 3).

### The placental weight at 13.5 GD and foetal weight at 16.5 GD were increased in *AQP1*^−/−^ mice

Next, we measured the wet weights of the foetus, placenta and foetal membrane as well as the area of the placenta in mice after AQP1 depletion at different stages of pregnancy. The foetal and foetal membrane weights and calculated placental areas did not differ among foetuses from 37 *AQP1*^−/−^ mice at 13.5 GD (*P* > 0.05, Table 2). However, the placental weight of the foetuses of 37 *AQP1*^−/−^ mice was higher than that of the foetuses of 40 *AQP1*^+/+^ littermates at 13.5 GD (*P* < 0.05, Table 2). At 16.5 GD, there were no significant differences in the foetal membrane, placenta weights or placental area between 41 *AQP1*^−/−^ and 42 *AQP1*^+/+^littermates (*P* > 0.05, Table 3). Interestingly, the weight of foetuses from 41 *AQP1*^−/−^ mice was increased compared with that of foetuses from 42 age-matched *AQP1*^+/+^ counterparts at 16.5 GD (*P* < 0.05, Table 3).

### Differential expression of AQP8 and AQP9 in the placenta and foetal membrane of *AQP1*^−/−^ mice

**Fig. 1:**
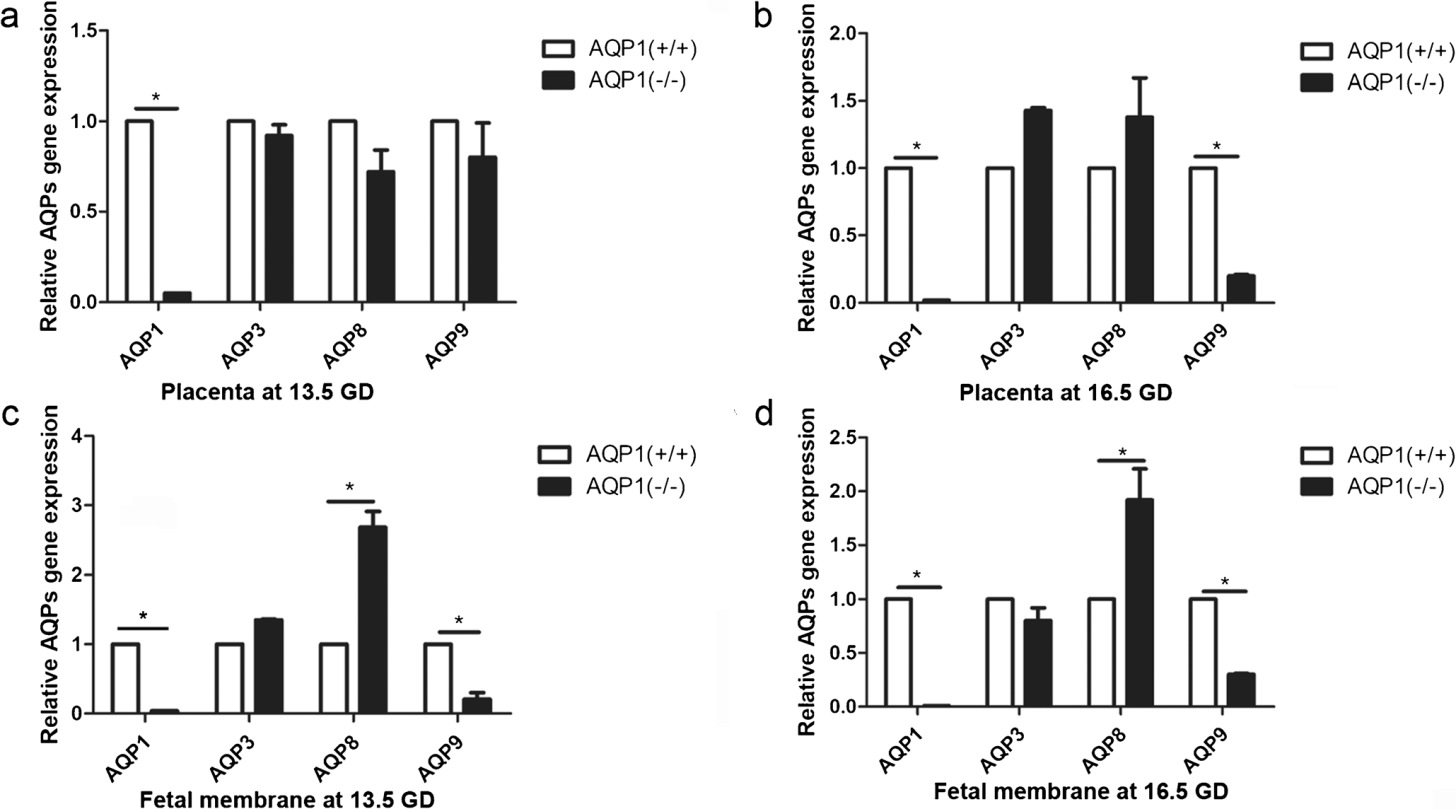
AQP1 depletion in mice causes a decrease of AQP9 mRNA in placenta and foetal membrane and an increase of AQP8 mRNA in foetal membrane. Relative mRNA expression of AQP1, AQP3, AQP8 and AQP9 was measured by qRT-PCR in placenta at 13.5 GD (a) and 16.5GD (b). Relative mRNA expression of AQP1, AQP3, AQP8 and AQP9 was detected using qRT-PCR in foetal membrane at 13.5 GD (c) and 16.5 GD (d). *P < 0.05 vs wild type mice

The expression of AQPs in the placentas of pregnant *AQP1*^−/−^ and *AQP1*^+/+^ mice at 13.5 GD and 16.5 GD was analysed at both the transcriptional and translational levels. As determined by quantitative reverse transcription polymerase chain reaction (qRT-PCR), AQP1 mRNA expression was not detected in the placentas of *AQP1*^−/−^ mice but was observed in *AQP1*^+/+^ mice at both 13.5 GD and 16.5 GD (Fig. 1a-b, Table 4). The placental mRNA levels of AQP3 and AQP8 were not significantly different between the two groups at both 13.5 GD and 16.5 GD (Fig. 1a-b, Table 4). Compared to the *AQP1*^+/+^ mice, there was no apparent difference in the placental mRNA expression of AQP9 at 13.5 GD in *AQP1*^−/−^ mice, but the AQP9 mRNA level in the placenta was lower in the *AQP1*^−/−^ mice at 16.5 GD (Fig. 1a-b, Table 4).

**Table 4:** Relative mRNA expression of AQP1, AQP3, AQP8 and AQP9 in placenta at 13.5 GD and 16.5 GD (mean ± SD)

Parameters	Cases	AQP1	AQP3	AQP8	AQP9
13.5 GD					
AQP1^+/+^	41	1	1	1	1
AQP1^−/−^	37	0.0512 ± 0.00124^*^	0.92 ± 0.0612	0.72 ± 0.122	0.80 ± 0.193
16.5 GD					
AQP1^+/+^	42	1	1	1	1
AQP1^−/−^	40	0.0214 ± 0.00262^*^	1.43 ± 0.0215	1.38 ± 0.291	0.201 ± 0.0147^*^

Data were analysed by ANOVA. *P < 0.05 versus wild type (*AQP1*^+/+^group)

*SD*, standard deviation; *ANOVA*, analysis of variance

**Fig. 2:**
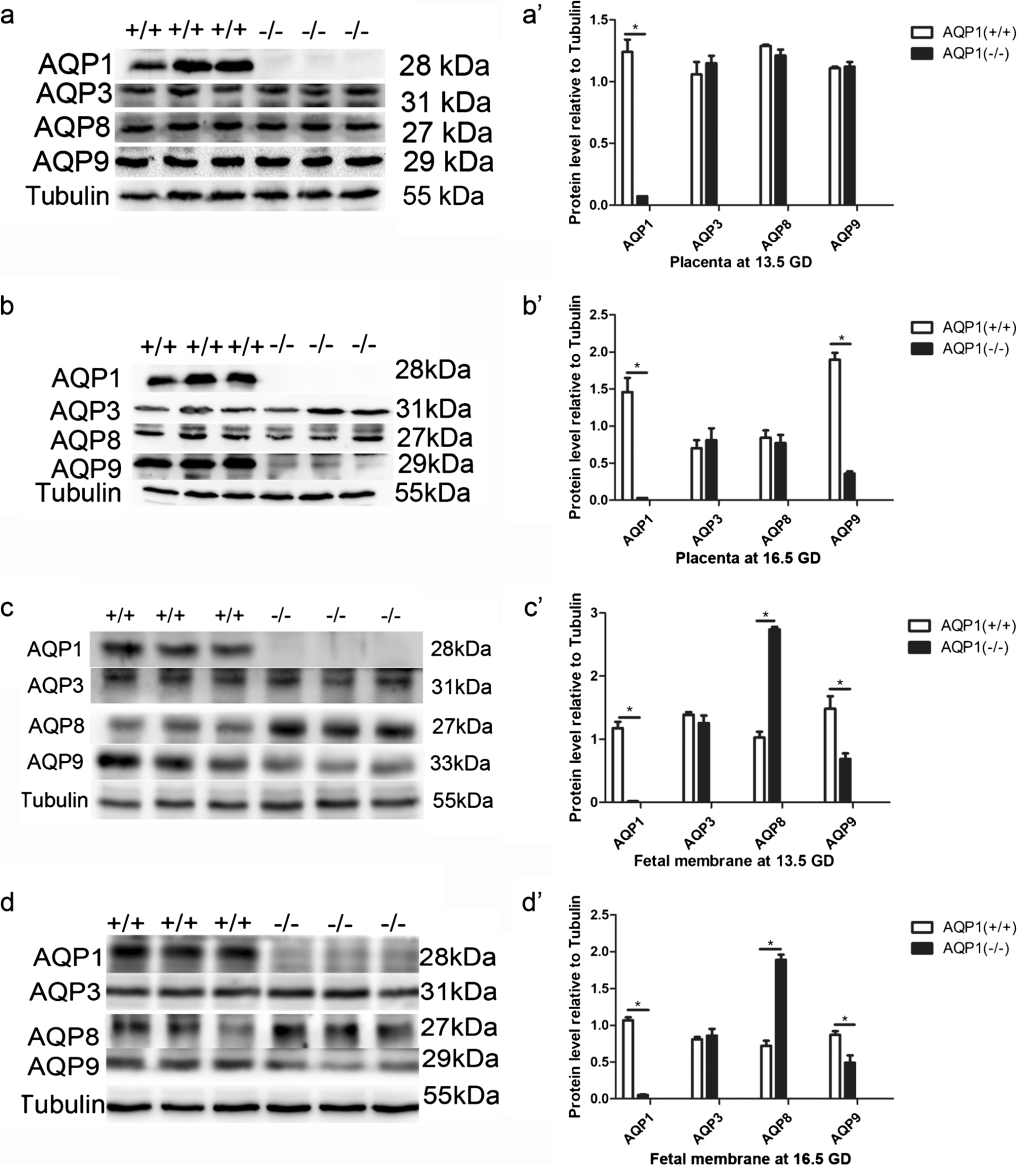
AQP1 knockout mice exhibit a decrease of AQP9 protein in placenta and foetal membrane and an increase of AQP8 protein in foetal membrane. Expression of AQP1, AQP3, AQP8 and AQP9 proteins was measured by western blotting in placenta at 13.5 GD (a–a′) and 16.5GD (b–b′). Expression of AQP1, AQP3, AQP8 and AQP9 proteins was determined by western blotting in foetal membrane at 13.5 GD (c–c′) and 16.5GD (d–d′). Quantitative data for each AQP protein were presented in the histogram (a′–d′)

The qRT-PCR results further demonstrated that AQP1 was not expressed in the foetal membranes of *AQP1*^−/−^ mice, unlike *AQP1*^+/+^ mice (Fig. 1c-d, Table 5). No significant difference in the mRNA expression of AQP3 in the foetal membrane was found between these two groups at 13.5 GD and 16.5 GD (Fig. 1c-d, Table 5). However, the mRNA level of AQP8 in the foetal membranes of the *AQP1*^−/−^ mice was significantly elevated compared with that of the *AQP1*^+/+^ mice (Fig. 1c-d, Table 5). Conversely, AQP9 mRNA expression was reduced in the foetal membranes of *AQP1*^*−/−*^ mice at both 13.5 GD and 16.5 GD (Fig. 1c-d, Table 5). Western blotting data validated that these changes in the mRNA expression of AQP1, AQP3, AQP8 and AQP9 in *AQP1*^−/−^ mice were consistent with changes in their proteins expression in the placenta and foetal membranes at 13.5 GD and 16.5 GD (Fig. 2a-d').

**Table 5:** Relative mRNA expression of AQP1, AQP3, AQP8 and AQP9 in foetal membrane at 13.5 GD and 16.5 GD (mean ± SD)

Parameters	Cases	AQP1	AQP3	AQP8	AQP9
13.5 GD					
AQP1^+/+^	41	1	1	1	1
AQP1^−/−^	37	0.0416 ± 0.00113^*^	1.35 ± 0.0128	2.68 ± 0.232^*^	0.212 ± 0.0932^*^
16.5 GD					
AQP1^+/+^	42	1	1	1	1
AQP1^−/−^	40	0.0123 ± 0.00137^*^	0.0814 ± 0.125	1.92 ± 0.293^*^	0.302 ± 0.0142^*^

Data were analysed by ANOVA. *P < 0.05 versus wild type (*AQP1*^+/+^group)

*SD*, standard deviation; *ANOVA*, analysis of variance

### AQP9 expression is decreased in AQP1 siRNA-transfected WISH cells

**Fig. 3:**
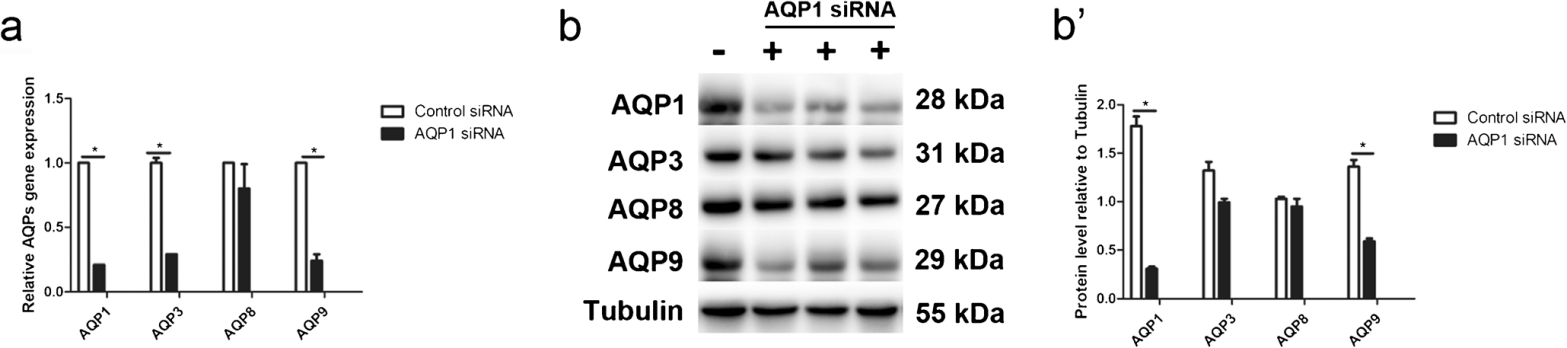
AQP9 expression is decreased in AQP1 siRNA transfected WISH cells. Relative mRNA expression of AQP1, AQP3, AQP8 and AQP9 was measured by RT-PCR in WISH cells after AQP1 siRNA transfection. *P* < 0.05 vs control siRNA transfection (a). Expression of AQP1, AQP3, AQP8 and AQP9 proteins was detected by western blotting in AQP1 siRNA transfected WISH cell (b). Quantitative data for control siRNA and AQP1 siRNA protein were presented in the histogram (b′)

To further confirm our findings in *AQP1*^−/−^ mice, WISH cells were transfected with 40 nM AQP1 siRNA. Compared with the control siRNA-transfected cells, AQP1 siRNA-transfected cells exhibited AQP1 mRNA and protein levels reduced by 79% and 81%, respectively (Fig. 3a-b', Table 6), and the mRNA and protein expression of AQP9 was significantly reduced (Fig. 3a-b', Table 6). Although the AQP3 mRNA expression was lower in AQP1 siRNA-transfected WISH cells (Fig. 3a, Table 6), AQP3 protein expression was not changed after AQP1 downregulation (Fig. 3b-b'). Furthermore, AQP1 downregulation did not alter the mRNA and protein levels of AQP8 in WISH cells (Fig. 3a-b', Table 6). These results demonstrate that AQP1 mainly regulates the expression of AQP9 in WISH cells.

**Table 6:** Relative mRNA expression of AQP1, AQP3, AQP8 and AQP9 in control siRNA WISH cell and AQP1 siRNA WISH cell (mean ± SD)

Parameters	AQP1	AQP3	AQP8	AQP9
Control	1	1	1	1
AQP1 siRNA	0.211 ± 0.001^*^	0.293 ± 0.002^*^	0.802 ± 0.191	0.244 ± 0.051^*^

Data were analysed by ANOVA. *P < 0.05 versus control group

*SD*, standard deviation; *ANOVA*, analysis of variance

### Expression of AQP1 is associated with AQP9 protein levels in oligohydramnios patients

**Fig. 4:**
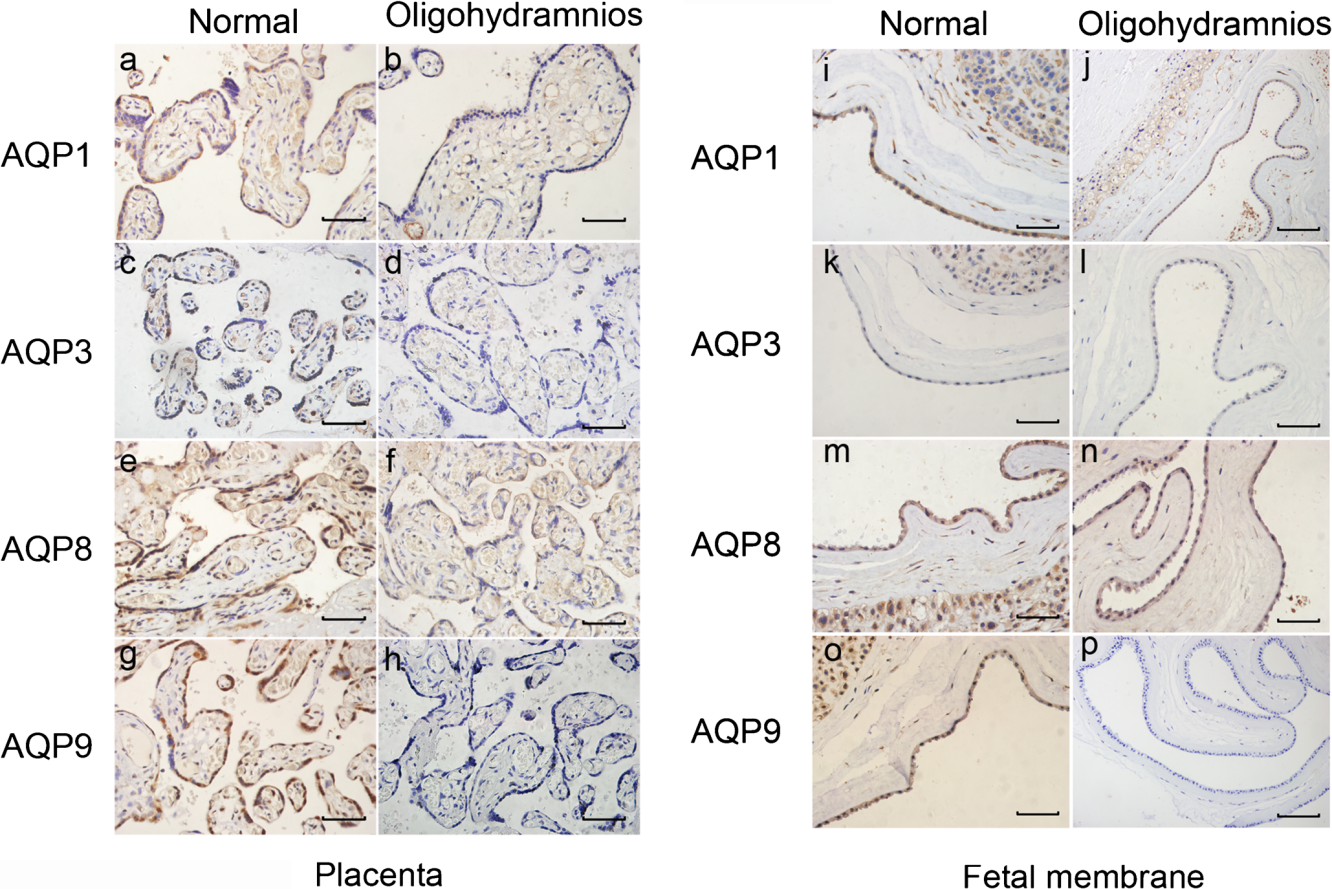
Expression of AQP1 is associated with AQP9 protein level in oligohydramnios patients. Immunohistochemical staining of AQP1, AQP3, AQP8 and AQP9 was performed in placenta in normal AF volume group and oligohydramnios group (a–h). Immunohistochemical staining of AQP1, AQP3, AQP8 and AQP9 was conducted in amnion epithelial cells and chorion in normal AF volume group and oligohydramnios group (i–p). Bar 50 μm

In this study, patients with oligohydramnios were chosen to determine whether the expression levels of AQP1 and other AQPs are correlated when AQP1 and other AQPs are decreased. The placenta and foetal membranes were immunostained with antibodies against AQP1, AQP3, AQP8 or AQP9 (Fig. 4a-p). AQP1 and AQP9 protein expression was detected in amnion epithelial cells, chorion cytotrophoblasts and placental trophoblasts (Fig. 4a, i, g, o). Compared with patients with a normal AFV, patients with oligohydramnios exhibited dramatically decreased AQP1 and AQP9 expression in the foetal membrane and placental trophoblasts (Table 7, * P* < 0.05, Fig. 4a-b, i-j, g-h, o-p). Additionally, the expression of AQP3 in placental trophoblasts from patients with oligohydramnios was dramatically decreased (Table 7, * P* < 0.05, Fig. 4c-d). No significant difference in AQP3 expression in the foetal membrane (Fig. 4k-l), or in AQP8 expression in placental trophoblasts and foetal membrane (Fig. 4e-f, m-n) was observed between the two groups (Table 7, *P* > 0.05). Moreover, among patients with a normal AFV, no significant difference in the expression of AQP1, AQP3, AQP8 or AQP9 was found in the placenta trophoblasts, amnion epithelial cells or chorion (Table 8 and Table 9, *P* > 0.05). In patients with oligohydramnios, AQP9 expression was positively associated with AQP1 expression in both placental trophoblasts (Table 8, *P* < 0.05, *r* = 0.640) and foetal membranes (amnion epithelial cells and chorion cytotrophoblasts) (Table 9, *P* < 0.05, *r* = 0.634).

**Table 7:** Expression of AQP1, AQP3, AQP8 and AQP9 protein in human placenta and foetal membranes in normal amniotic groups and oligohydramnios groups by immunohistochemistry (mean ± SD)

Parameters	Cases	AQP1 (%)	AQP3 (%)	AQP8 (%)	AQP9 (%)
Placenta					
Normal	15	87.2 ± 23.1	72.3 ± 11.3	90.1 ± 21.2	88.2 ± 90.1
Oligohydramnios	15	56.2 ± 14.2^*^	54.2 ± 19.4^*^	88.2 ± 15.4	14.2 ± 1.34^*^
Foetal membranes					
Normal	15	89.2 ± 20.1	86.2 ± 13.4	98.2 ± 13.3	92.1 ± 6.12
Oligohydramnios	15	60.2 ± 18.3^*^	78.2 ± 22.4	90.1 ± 21.4	11.9 ± 1.14^*^

Data were analysed by ANOVA. *P < 0.05. vs normal amniotic group

*SD*, standard deviation; *ANOVA*, analysis of variance

**Table 8:** The relevance among AQP1 and AQP3, AQP8, AQP9 in placenta trophoblasts of normal group and oligohydramnios group

		AQP1				AQP1	
Normal	*n*	*r*	*P*	Oligohydramnios	*n*	*r*	*P*
AQP3	15	0.401	0.202	AQP3	15	0.171	0.582
AQP8	15	0.342	0.393	AQP8	15	0.0833	0.801
AQP9	15	0.284	0.403	AQP9	15	0.640	0.018^*^

Data were analysed by Pearson correlation analysis, *P < 0.05

**Table 9:** The relevance among AQP1 and AQP3, AQP8, AQP9 in foetal membranes of normal group and oligohydramnios group

		AQP1				AQP1	
Normal	*n*	*r*	*P*	Oligohydramnios	*n*	*r*	*P*
AQP3	15	0.352	0.291	AQP3	15	− 0.542	0.171
AQP8	15	− 0.265	0.381	AQP8	15	0.245	0.482
AQP9	15	0.548	0.0650	AQP9	15	0.634	0.0214^*^

Data were analysed by Pearson correlation analysis, *P < 0.05

## Discussion

AQPs are distributed throughout the male and female reproduction systems, where they facilitate gamete transport, fertilization and early embryo development (Skowronski et al. 2009; Zhang et al. 2012a). An increase in embryo numbers was observed in *AQP8*^−/−^ mice (Sha et al. 2011). Consistently, female *AQP8*^−/−^ mice displayed elevated fertility due to an increase in follicular maturation and ovulation via reduced granulosa cell apoptosis (Su et al. 2010). In contrast, pregnant *AQP4*^−/−^ mice displayed reduced fertility and a lower pregnancy rate and litter size. This may be caused by the presence of smaller ovaries containing fewer antral follicles and corpora lutea and a decreased uterine response to gonadotropins in *AQP4*^−/−^ mice compared to wild-type mice (Sun et al. 2009). In the male reproductive system, water and solute movement play a vital role in balancing the intracavitary environment for development and maturation of the male reproductive system. AQP1 has been observed in the reproductive tracts of male mammals (Huang et al. 2006). The dysregulation or knockdown of AQP1 might lead to male reproductive system disorders. Consistently, AQPs are also distributed in female reproductive tissues. AQP1 is primarily distributed in the vagina, uterus, oviduct, granulosa cells and oocytes. The main function of AQP1 is tubal fluid transport, and AQP1 plays an important role in maintaining the water homeostasis of the oviduct and uterus and facilitates the transport and fertilization of gametes (Gannon et al. 2000). Impairment of AQP1 can lead to decreased fertility in both males and females. In the present study, we revealed a decreased copulation plug rate and successful pregnancy rate in *AQP1*^−/−^ mice, demonstrating that AQP1 is involved in regulating the function of the female reproductive system. Nevertheless, the number of macroscopic atrophic embryos in *AQP1*^−/−^ mice and *AQP1*^+/+^mice did not differ at either 13.5 GD or 16.5 GD, suggesting that the AQP1 protein does not affect embryogenesis and development. Thus, further studies should be carried out to specifically elucidate the underlying molecular mechanism of reduced female reproductive performance due to the absence of AQP1 and cellular events responsible for the effect.

In our previous study, AQP1 was detected in placental vascular endothelial cells (Zhu et al. 2009), which may reflect a role in angiogenesis. Herein, we found that the placenta weight of *AQP1*^−/−^ mice was dramatically higher than that of *AQP1*^+/+^ counterparts at 13.5 GD, and the embryonic weight of in *AQP1*^*−/−*^ mice was higher than that of *AQP1*^+/+^ mice at 16.5 GD. In support of the role of AQP1 in angiogenesis, the placentas of *AQP1*^−/−^ mice displayed an altered blood vessel structure, an increased number of trophoblast cells and nodules and enhanced nucleated red blood cell counts (Zheng et al. 2014). Therefore, we speculate that the placentas of *AQP1*^−/−^ mice exhibit abnormal angiogenesis that results in hyperplasia and oedema. Indeed, an increased AFV and larger placentas were observed in *AQP1*^−/−^ mice at 16.5 GD, which might create a perfect environment for foetuses in utero and more nutrients to increase foetal weight. Our findings indicate that AQP1 has a vital function in placental and foetal growth.

The foetuses of homozygous *AQP1*^−/−^ mice exhibit polyhydramnios (Zheng et al. 2014). The degenerating placentas of *AQP1*^−/−^ displayed compressive deformation of the blood vessel structure and an increased number of syncytiotrophoblast nodules. Additionally, the permeability of trophoblast cells in AQP1^−/−^ mice was shown to be significantly decreased (Sha et al. 2015). Consistent with these results, our data suggested that female *AQP1*^−/−^ mice serve as a model of polyhydramnios. Furthermore, polyhydramnios was observed at 16.5 GD rather than 13.5 GD, which is consistent with the occurrence of polyhydramnios in late gestation in humans (Brace 1997). In the latter half of gestation, AF is largely produced from foetal urine and foetal lung secretions. The AF is primarily reabsorbed through foetal swallowing, but the intramembranous pathway also accounts for some AF removal. Several possible mechanisms for the increase in AF volume have been reported (King et al. 1996). Although AQP1 has been detected in pulmonary microvascular endothelial cells and pneumocytes, AQP1 deletion did not influence the production or clearance of alveolar fluid (Verkman et al. 2000, Chou et al. 1999). Furthermore, *AQP1*-deficient mice showed impaired absorption of renal fluid and the inability to concentrate urine (Chou et al. 1999, Verkman 1999), which may increase urinary flow to the amniotic cavity, suggesting the important role of the AQP1 protein in the kidney. On the other hand, when AF absorption is decreased, AQP1 may participate in regulating the movement of water in the foetal membranes and placental trophoblast (Zheng et al. 2014), while AQP1 deficiency reduces the fluid resorption capacity. The composition of the AF was not changed in *AQP1*^−/−^ mice at 16.5 GD; thus, we speculated that the observed polyhydramnios and decreased AF osmolality were associated with AQP1 (Mann et al. 2002). Changes in the AF volume may be due to alterations in AF osmolality caused by AQP1-mediated water transport.

Specifically, AQP1, AQP3, AQP8 and AQP9, which play a vital role in maternal-foetal fluid exchange homeostasis, have been detected in the placenta and foetal membranes (Beall et al. 2007). Compensatory changes in the expression of other AQPs induced by alterations in AQP1 expression were also observed in this study. While AQP8 was upregulated in the foetal membrane, the expression of AQP9 was reduced in foetal membranes and placenta of *AQP1*^−/−^ mice. Hence, upregulation of AQP8 in the foetal membranes after AQP1 depletion may lead to polyhydramnios through a compensatory mechanism, while the expression of AQP9 in the placenta and foetal membranes is decreased after *AQP1* depletion. This phenomenon might be related to the different classifications and biological functions of AQPs. AQP1 and AQP3 play a vital role in passive water movement across the amnion (Damiano 2011) and other studies suggest that AQP8 and AQP9 are fundamental to the regulation of foetal water and solute flow through both intramembranous absorption and placental water transfer from mother to foetus (Wang et al. 2004; Wang et al. 2001). AQP1 and AQP8 are characterized as classical AQPs selectively permeable to only water (Ishibashi et al. 2011), whereas AQP9 functions as an aquaglyceroporin permeable to water, reactive oxygen species, non-polar solutes, metalloids and gases (Madeira et al. 2015; Mukhopadhyay et al. 2014). AQP3 mRNA expression was lower in the AQP1 siRNA-transfected WISH cells, while its protein level was unchanged; this discrepancy between AQP3 protein and mRNA expression is mostly likely the result of biology of gene expression and the regulation of protein synthesis at various levels, such as the posttranscriptional, translational or and posttranslational levels (Tian et al. 2004).

Furthermore, we explored the association between AQP1 and other AQPs in human WISH cells, and found that inhibition of AQP1 expression significantly reduced AQP9 expression. In our previous study (Zhu et al. 2009; Jiang et al. 2012), AQP1 and AQP9 expression in the amnion was decreased in pregnancies with isolated oligohydramnios, so pregnancies with oligohydramnios and a normal AF volume were selected to establish the correlation between AQP1 and AQP9 protein expression. Our results showed that the expression of AQP1 and AQP9 was dramatically decreased in both placental trophoblasts and amnion epithelial cells in oligohydramnios, which suggested that downregulation of AQP1 and AQP9 is an adaptive response to isolated oligohydramnios, which leads to a reduction in intramembranous absorption, maintaining AF homeostasis. On the other hand, AQP1 and AQP3, AQP8 and AQP9 expressions levels in placenta trophoblasts, amnion epithelial cells and the chorion were not significantly correlated in normal pregnant women. However, in oligohydramnios patients, AQP9 expression in the placenta and foetal membranes was positively correlated with AQP1 expression in both placental trophoblasts and amnion epithelial cells.

In summary, while the water channel AQP1 may influence pregnancy outcome and participate in the regulation of AF volume and osmotic pressure, it does not affect embryogenesis and development. In addition, changes in AQP1 may lead to compensatory alterations in the expression of other AQPs in both the placenta and foetal membranes. Further studies should be carried out to clarify this compensatory mechanism in the future.
